# Reimbursement categories as a way of allocating measures and monitoring expenditures on medicines

**DOI:** 10.1186/2052-3211-8-S1-P24

**Published:** 2015-10-05

**Authors:** Aneta Lipińska, Maciej Pomorski, Wojciech Matusewicz, Katarzyna Jagodzińska-Kalinowska

**Affiliations:** 1 Agency for Health Technology Assessment and Tariff System, Warsaw, 02-611, Poland

## Background

Control of expenditure on reimbursement of medicines distributed to patients through pharmacies poses many problems despite it providing easy access to the drugs. Other reimbursement categories, chemotherapy drugs and drug programs, may allow health authorities to allocate measures and monitor expenditures under the whole budget for drugs.

## Objectives

The aim of this publication is to describe two reimbursement categories in Poland, chemotherapy drugs and drug programs, and to assess strengths and weaknesses of them from the payer's and patients' perspective.

Policies targeted: The abstract presents evaluation of reimbursement categories which interface between out-patient and in-patient sectors, and influence of these reimbursement categories on public payer, health care providers (HCP), and patients.

## Methods

This work is a description of a model implemented in Poland and an evaluation of current (up to 28 May 2015) policy in terms of rationalizing and controlling expenditures and access to health care services. The study examines the public sector and is related to the out-patient as well as in-patient sectors because of the mixed nature of these solutions.

## Results

The essence of the solution implemented in Poland is agreements between the payer (National Health Fund (NHF)) and individual providers, but what is important is that neither the NHF nor HCP are obligated to enter into agreement. Medicines in drug programs or chemotherapy drugs are distributed by hospitals or on an out-patient basis. Their consumption is reported in detail to the NHF. Drug programs ensure that the qualifying criteria are the same, the monitoring of therapy effects is simplified and the NHF can control if it pays for the successful treatment. In general, the NHF is knowledgeable about the availability of services and maximum expenditures. On the other hand, adherence to the limit and freedom in concluding contracts may restrict the availability of services (queues, migration of patients in search of treatment) and burden patients with additional costs. Due to fixed exclusion criteria in drug programs there is a possibility that cessation of therapy may harm patients more than unsuccessful treatment.

## Conclusions

Distribution of therapies to the patients within assessed categories limits access – only through contracted HCP (hospital and ambulatory care), but on the other hand it rationalizes the use of medicines. Careful analysis of health needs and use of incentives for health care providers may allow a proper allocation of resources and ensuring patients an equal access to services.

